# Release of Cationic Drugs from Charcoal

**DOI:** 10.3390/ma12040683

**Published:** 2019-02-25

**Authors:** Chiara Di Ruocco, Maria Rosaria Acocella, Gaetano Guerra

**Affiliations:** Department of Chemistry and Biology and INSTM Research Unit, Università di Salerno, via Ponte don Melillo, 84084 Fisciano (SA), Italy; c.diruocco1@studenti.unisa.it (C.D.R.); rosyaco@hotmail.it (M.R.A.)

**Keywords:** wide angle X-ray diffraction, infrared spectra, pH sensitive ion release

## Abstract

The goal of this research is to improve preparation of charcoal adducts in a manner suitable for cationic drug release, possibly using an eco-friendly procedure. Charcoal, widely commercialized for human ingestion, is oxidized by hydrogen peroxide in mild conditions. Adducts of a cationic drug (lidocaine hydrochloride, a medication used as local anesthetic) with charcoal are prepared after basification of charcoal and characterized mainly by elemental analysis, wide-angle X-ray diffraction, infrared spectroscopy and thermogravimetry. The drug in the prepared adducts is present in amount close to 30% by weight and can be readily released to both neutral and acidic aqueous solutions. Cation release, as studied by UV spectra of aqueous solutions, is faster in acidic solutions and is faster than for adducts with graphite oxide, which can be prepared only in harsh conditions.

## 1. Introduction

Cationic amphiphilic drugs and antibacterials constitute one of the largest classes of pharmaceutical compounds [1,2,3,4,5,6,7,8,9,10]. 

Different systems for controlled release of cationic drugs are used. Cationic drugs may be bound to anionic polymeric exchange resins (i.e., to a polyelectrolyte), which are generally used after incorporation into liquids or tablets [11]. Sensitive cationic drugs can also be included in delivery systems based on pH-responsive nanogels, to improve their stabilization and control their release [12].

The release of antibacterial cations from intercalation and exfoliation compounds of layered structures, like clays [13,14,15,16,17,18] and graphite oxide (GO) [19,20,21,22,23] has also been suggested. The application of clays as carrier of antibacterial quaternary ammonium or phosphonium salts is limited by their bad dispersion in water [17,18]. Applications of graphite oxide [19,20,21,22,23] or of oxidized carbon black [24] as cationic drug carriers is possibly limited by the harsh oxidations procedures needed for preparation of intercalation or exfoliation compounds [19,20,21,22,23,24]. 

In this paper, we describe an eco-friendly procedure leading to adducts of cationic drugs with charcoal, which are possibly suitable for cationic drug release. In this respect, it is worth noting that charcoal is widely used to treat human flatulence, indigestion and diarrhea, without professional prescription. 

In particular, we describe oxidation of charcoal by hydrogen peroxide in mild conditions and preparation of compounds of oxidized charcoal with lidocaine hydrochloride, which is a medication used as local anesthetic [25,26]. Release of lidocaine, from polymer gels [27,28,29,30,31] as well as from liquid crystal structures [32] and polymer threads [33], has been widely studied in the literature. Presently, release of this cationic drug in neutral and acid aqueous solutions from adducts with GO and oxidized charcoal are compared.

## 2. Experimental

### 2.1. Materials, Oxidation Procedures and Preparation of Compounds

Charcoal (Carbo lignis pulveratus) was from Caesar & Loretz GmbH (Hilden, Germany). Its Oxygen/Carbon weight ratio, as evaluated by elemental analysis, was high (O/C = 0.29). Lidocaine hydrochloride monohydrate was supplied by Sigma-Aldrich (Milano, Italy).

Oxidation of charcoal was conducted by hydrogen peroxide at 60 °C. In a typical procedure, 1 mL of H_2_O_2_ 30 wt.% was used for 2 mg of charcoal. The O/C ratio progressively increased with the treatment time, reaching 0.7 after 24 h. Most of the reported results refer to charcoal oxidized for 24 h. A similar oxidation procedure has been used to achieve edge oxidation of graphite platelets [34].

GO samples were prepared by Hummers’ method [35] from high surface area graphite [36] (Synthetic Graphite 8427^®^, from Asbury Graphite Mills Inc. (Asbury, NJ, USA), with a minimum carbon wt.% of 99.8 and a surface area of 330 m^2^/g. 

Both oxidized charcoal and GO were basified before preparation of their compounds with the cationic drug. In particular, powders (100 mg) were dispersed in 0.05 M NaOH solution (20 mL) and stirred for 15 minutes. Lidocaine aqueous solution (100 mL) was added in this dispersion and the reaction mixture was stirred at room temperature for 1h. The reported results refer to the charcoal/lidocaine with a weight ratio of 1/3. Similar results were obtained by operating in excess of lidocaine. The slurry was washed with deionized water and dried at 60 °C for 12 h. 

### 2.2. Characterization Methods

Elemental analyses were performed with a Thermo FlashEA 1112 Series CHNS-O analyzer by Thermo Fisher Scientific Inc. (Waltham, MA, USA), after pretreating samples in an oven at 100 °C for 12 h.

Surface areas of the used carbon materials were measured by nitrogen adsorption at liquid nitrogen temperature (77 K) with a Nova Quantachrome 4200e instrument by Quantachrome Instruments (Boynton Beach, FL, USA). Before adsorption measurements, samples were degassed at 60 °C under vacuum for 24 h. The surface area (SA_BET_) values were determined by using 11-point Brunauer-Emmett-Teller (BET) analysis.

Thermogravimetric analyses (TGA) were carried out on a TG 209 F1, manufactured by Netzsch Geraetebau GmbH (Selb, Germany), from 20 to 800 °C at a heating rate of 10 °C, under N_2_ flow.

Wide-angle X-ray diffraction (WAXD) patterns were obtained in reflection, at 35 kV and 40 mA, using the nickel filtered Cu-Kα radiation (1.5418 Å), by an automatic Bruker D8 Advance diffractometer (Karlsruhe, Germany). 

FTIR spectra were obtained at a resolution of 2.0 cm^−1^ with a FTIR spectrometer (Bruker Vertex70, Bruker, Karlsruhe, Germany) equipped with deuterated triglycine sulfate (DTGS) detector and a KBr beam splitter, using KBr pellets. The frequency scale was internally calibrated to 0.01 cm^−1^ using a He-Ne laser. 32 scans were signal averaged to reduce the noise.

UV-vis spectra were recorded using a Perkin Elmer Lambda 800 UV-vis spectrophotometer (Waltham, MA, USA).

The released amount of lidocaine from adducts with charcoal and GO, in neutral (1 wt.% of NaCl) and acid (0.05 M of HCl) aqueous solutions was measured as a function of the soaking time. A sealed cellulose filter (“Macherey-Nagel MN226”, Düren, Germany), containing 50 mg of powder compounds, was soaked in a flask with 0.1 L of aqueous solution at room temperature, maintaining the system constantly stirred. Aliquots of the transparent aqueous solution were sampled by syringe after different times and the concentrations of the released lidocaine were measured by using an UV-vis spectrophotometer.

## 3. Results and Discussion

### 3.1. Oxidation of Charcoal by Hydrogen Peroxide

The used charcoal has the composition shown in Table 1, as determined by elemental analysis. The observed O/C weight ratio was definitely higher than for carbon black samples, being generally lower than 0.1. Its surface area was rather low: SA_BET_ ≈ 50 m^2^/g.

**Figure 1:**
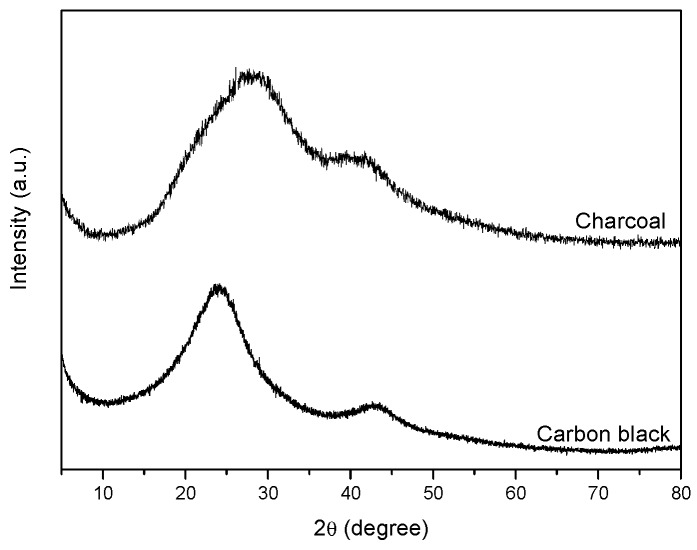
Wide-angle X-ray diffraction (WAXD) patterns of the used charcoal and of a typical carbon black sample.

The X-ray diffraction pattern of charcoal (upper curve of Figure 1) showed two very broad amorphous halos, broader than those observed for carbon black samples (the pattern of a carbon black with a surface area of 150 m^2^/g is shown, for comparison, as the lower curve in Figure 1) [24]. Diffraction halos of carbon black were interpreted by a disordered spatial arrangement of defective graphene layers, exhibiting short in-plane correlation length (typically in the range of 2–3 nm), as confirmed by the ability of oxidized carbon black (oCB) to form ordered intercalation compounds, with ammonium cations [24]. The broader amorphous halos of charcoal clearly indicated the occurrence of a higher degree of disorder associated with its high oxygen content.

Irrespective of its high oxygen content, the used charcoal after basification was only able to bind small amounts of cations; e.g., the content of bound cationic lidocaine is lower than 2 wt.%. Hence, to get compounds with significant cation content, higher degrees of oxidation were needed. However, due to the low degree of structural order, mild oxidation conditions were sufficient to get degrees of oxidation suitable for adduct formation with cations. In particular, oxidation at 60 °C by hydrogen peroxide (30 wt.%) is sufficient to achieve O/C weight ratios as high as 0.7 (Table 1), analogous to those obtained with high-surface-area graphite by the harsh Hummers’ method. It is also worth adding that for oxidation of carbon black by Hummers’ method, O/C weight ratios higher than 0.6 are only reached for carbon black samples with a surface area higher than 120 m^2^/g [24]. Hence the substantial oxidation of charcoal already by hydrogen peroxide was not due to its surface area (definitely low, SA_BET_ ≈ 50 m^2^/g) but rather to its defective structure associated with its high starting oxygen content.

**Figure 2:**
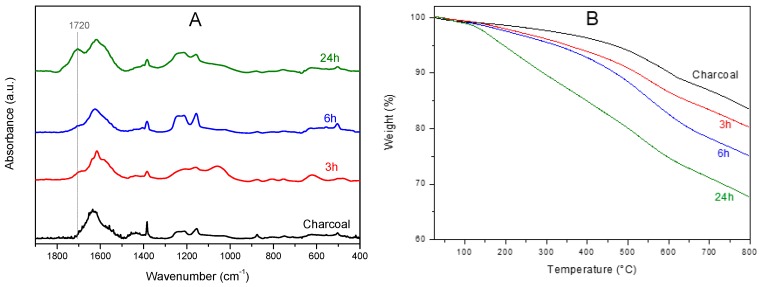
(**A**) FTIR spectra and (**B**) TGA scans of the used charcoal sample after different times of oxidation by H_2_O_2_ at 60 °C.

The charcoal oxidation was clearly apparent by FTIR spectra, like those of Figure 2A. As a consequence of oxidation, an absorbance peak at 1720 cm^−1^ appears that becomes very intense after 24 h of treatments, clearly indicating the formation of carboxylic groups. An increase of intensity of the band in the range 1270–1120 cm^−1^ was also observed, with two peaks centered at 1155 cm^−1^ and 1215 cm^−1^, which are possibly due to phenol C–O groups [37,38]. 

The charcoal oxidation was also associated with large changes in the thermogravimetric behavior, as clearly apparent by TGA scans shown in Figure 2B. In fact, as the O/C ratio increased the degradation phenomena were shifted toward lower temperatures.

### 3.2. Formation of Adducts between Oxidized Charcoal and Organic Cations

For oxidized CB, adduct formation procedures with tetraalkylammonium cations exhibiting two long hydrocarbon tails (2HT) lead to intercalated compounds [24]. This was clearly shown by the appearance, for samples with O/C > 0.5, of well-defined diffraction peaks that can be rationalized as 001, 003 and 005 reflections of an oCB/2HT intercalate structure, with a periodicity of *d* = 4.8 nm (Figure 6 in Reference [24]).

The same procedure, when applied to oxidized charcoal, also leads to formation of adducts, as proved by the occurrence of cation exchange and weight increase. However, no crystalline peaks appeared indicating that no intercalate structure was formed. This clearly confirms the presence in charcoal of a definitely higher degree of disorder, relative to CB, as suggested by the diffraction halos of Figure 1.

Neither oCB or oxidized charcoal were able to form intercalate adducts with lidocaine and in both cases only disordered adducts were obtained. 

**Figure 3:**
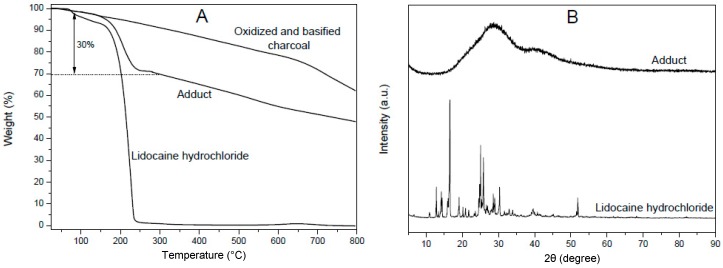
TGA scans (**A**) and WAXD patterns (**B**) of lidocaine hydrochloride and of its adducts with oxidized and basified charcoal (charcoal/lidocaine, 70/30 wt/wt), as prepared by a large excess of lidocaine.

A TGA scan of the lidocaine adduct with oxidized charcoal (O/C, wt/wt = 0.7), as prepared in the presence of a large excess of the organic salt (C/lidocaine weight ratio of 1/3), is shown in Figure 3A. A comparison with TGA of the starting basified charcoal (upper scan in Figure 3A) and of lidocaine hydrochloride (lower scan in Figure 3A) shows that the obtained adduct presented a lidocaine content close to 30 wt.%. 

The X-ray diffraction pattern of this adduct is shown in Figure 3B and presents only the broad diffraction halo of charcoal. A comparison with the WAXD spectrum of lidocaine hydrochloride (bottom patterns in Figure 3B) clearly shows that the crystallinity of lidocaine hydrochloride was completely lost as a consequence of adduct formation. A similar behavior was observed for adducts of lidocaine hydrochloride with GO.

### 3.3. Lidocaine Release from Adducts with Oxidized Charcoal and Graphite Oxide 

**Figure 4:**
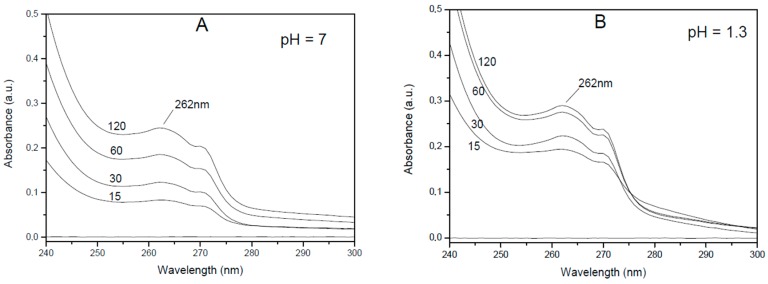
UV spectra in the 240−300 nm range of aqueous solutions, after different soaking times (from 15 min up to 120 min) of the lidocaine adduct with oxidized charcoal: (**A**) neutral saline solution; (**B**) acidic (pH = 1.3) solution.

The obtained adducts of lidocaine with oxidized charcoal and graphite oxide presented similar large lidocaine content (30 wt.% and 33 wt.%, respectively). These adducts were compared for their kinetics of release of the ionic drug in aqueous solutions, neutral (pH = 7 with 1 wt.% of NaCl) and acid (pH = 1.3, HCl 0.05 M), roughly corresponding to the pH of a human colon and stomach, respectively. The released amounts were evaluated by UV measurements in the spectral range 240–300 nm on the solutions, as shown for adducts with oxidized charcoal in Figure 4. 

**Figure 5:**
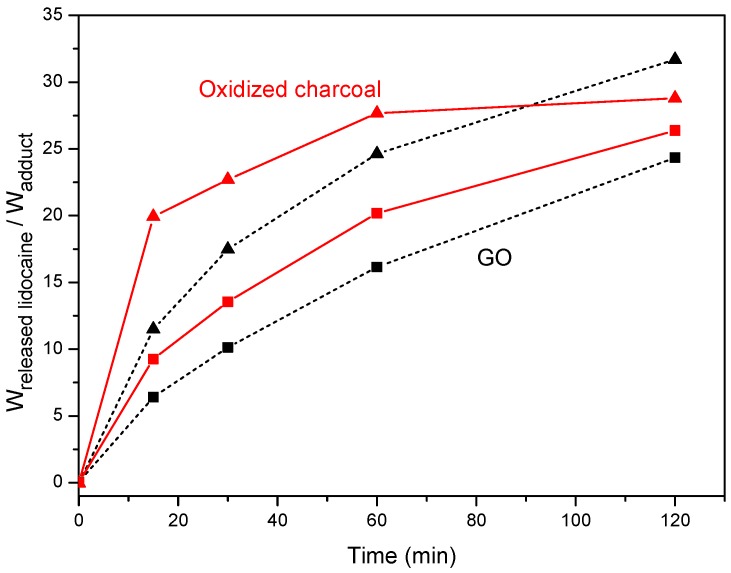
Lidocaine release from its adducts with oxidized charcoal (red continuous curves) and with graphite oxide (GO) (black dashed curves), in neutral (squares) and acidic (pH = 1.3; triangles) solutions.

The released amount of lidocaine from adducts with charcoal (red continuous curves) and GO (black dashed curves), in neutral and acidic (pH = 1.3) aqueous solutions are reported versus the soaking time in Figure 5.

The data of Figure 5 show that the release of the ionic drug in the aqueous acid solution was complete, for adducts with oxidized charcoal and oxidized graphite, after 1 h and 2 h, respectively. For both neutral and acidic solutions, the cation release was faster for the charcoal adduct than for the GO adduct.

## 4. Conclusions

An eco-friendly procedure leading to adducts of a cationic drug with charcoal, which is suitable for cationic drug release, is presented in this study. The proposed procedure includes oxidation by hydrogen peroxide in mild conditions of charcoal, which is widely commercialized for human ingestion without professional prescription. 

The oxidation procedure is followed by preparation of oxidized charcoal adducts with lidocaine hydrochloride (a medication used as local anesthetic). For oxidized charcoal with an O/C weight ratio of 0.7, a typical content of the drug in the prepared adducts is close to 30% by weight, nearly independent of the used excess of the drug. 

Release behaviors of this cationic drug in neutral and acid aqueous solutions from lidocaine adducts with GO and oxidized charcoal are compared. The release of the ionic drug in the aqueous acid solution is complete after 1 h. Both for neutral and acidic solutions the cation release is faster for the charcoal adduct than for the graphite oxide adduct.

The presently proposed cationic drug release system, with respect to other already known systems based on intercalation and exfoliation compounds of layered structures, has the advantage of being based on a solid substrate traditionally used for human ingestion, modified by a simple and eco-friendly procedure.

## Figures and Tables

**Table 1:** Elemental analysis of charcoal after different times of oxidation by H_2_O_2_. The last column indicates the water content as determined by thermogravimetry.

Charcoal	%N	%C	%H	%O	O/C	%H_2_O
Untreated	0.3	74.8	4.2	20.7	0.29	4.0
H_2_O_2_ oxidized, 3 h	1.0	68.8	1.9	28.2	0.41	5.6
H_2_O_2_ oxidized, 6 h	1.0	67.4	1.8	29.8	0.44	6.1
H_2_O_2_ oxidized, 24 h	0.9	57.2	1.5	40.4	0.70	7.6
